# Masticatory Efficiency of Thermoplastic Versus Conventional Acrylic Resin Mandibular Implant Overdenture With Bar/Clip Attachment

**DOI:** 10.1155/ijod/4212059

**Published:** 2025-04-17

**Authors:** Hamada Z. Mahross Atia, Ehab M. Atito, Mamdouh M. Mansour, Mohamed S. Osman, Wesam E. Badr, Mohamed A. Quassem

**Affiliations:** ^1^Removable Prosthodontics Department, Faculty of Dental Medicine, Al-Azhar University, Cairo, Egypt; ^2^Department of Substitutive Dental Sciences, College of Dentistry and Pharmacy, Buraydah Private Colleges, Buraydah 51418, Saudi Arabia

**Keywords:** bar/clip attachment system, masticatory efficiency, thermoplastic overdenture

## Abstract

**Objectives:** To evaluate the effects of different base materials of mandibular implant-supported overdenture with bar/clip attachment on masticatory efficiency.

**Materials and Methods:** Twenty-four edentulous patients were selected to place two interforaminal mandibular implants with custom-made cast bars to retain overdentures made from two different base materials and divided into two groups: Group I was the patients with heat-cured acrylic resin overdentures, and Group II was the patients with thermoplastic base overdentures. Masticatory efficiency was tested using three food categories (carrots, bananas, and apples) and measured by four parameters. One-way analysis of variance (ANOVA) with a post hoc Tukey test was used at the chosen probability level *p* < 0.05.

**Results:** Group II was more efficient than Group I with the carrots as the difference was statistically significant for two parameters: the number of chewing strokes until the mouth was free of food with *p*=0.018 and the number of swallows it takes for the mouth to be empty of food *p* ≤ 0.001. With apples, the significance was related to two parameters: how many chews there were before the first swallow *p*=0.053 and the number of swallows until the mouth was empty of food *p* ≤ 0.001. With bananas, the significance is related to two parameters, the number of swallows until the mouth was free of food with *p* ≤ 0.001 and the time in seconds until the mouth was free from food *p*=0.037.

**Conclusions:** The overdentures made with the thermoplastic base material and supported with bar/clip attachments were more masticatory efficient than patients whose overdentures were made of the traditional acrylic resin.

**Trial Registration:** This clinical study presentation has been registered and publicly figured at clinical trial.gov PRS for protocol registration and results system under ID NCT05877092 University and conducted following the CONSORT checklist.

## 1. Introduction

In the case of mandibular completely edentulous patients, implant overdentures were considered the primary treatment option over traditional dentures, especially when considering the extremes of denture stability, retention, masticatory performance, and patient satisfaction [1–3].

Although heat-cured acrylic resin material was conventionally applied as a denture base material for implant-supported overdentures, the thermally cured type with a flexible base is considered more efficient because it has a cushioning effect during the functional movement with the denture to distribute and dissipate the stresses arising throughout functional and parafunctional activities. However, the denture stability was enhanced through a tight fit of the underlying tissues and the application of undercuts with the flexible type of denture base material [3, 4].

Implant placement to retain mandibular overdentures was the typical method for enhancing the masticatory function and obtaining patient satisfaction with the mandibular edentulous ridge. According to various research studies, mandibular implant overdentures improve a patient's bite force with the ability to chew with required fewer chewing cycles and successfully consume tough foods compared with traditional dentures [5–8].

Regarding the attachment methods used with implant-supported removable overdentures, several methods are employed effectively with the ball and socket or bar-type attachments. However, the bar-type attachment that acts as a splint was considered a prim anchoring method for enhancing stresses and biomechanics dispersion. In addition, it increases the overdentures' stability and reduces the overloading danger of every implant through its large surface area with the rectification of implant non-parallelism cases [9–12]. Moreover, for the cases of an edentulous patient's rehabilitation, the computer aided design/computer-aided manufacturing (CAD/CAM) titanium bar-supported overdenture can be considered an important treatment choice [13].

Clinically, many tests were applied to evaluate the masticatory abilities and degradation level of foods like peanuts, carrots, and bananas, or synthetic substances like Optosil and Optocal. Moreover, there are several methods to evaluate the efficiency of mastication, such as the evaluation of mandibular border motions while eating, chewing, swallowing, jaw muscular functions, peak biting intensity, and serum plasma concentrations of homocysteine, vitamin B12, vitamin B6, albumin, blood folate, and creative protein levels [14–17].

The null hypothesis tested in this trial is that there is no difference in the masticatory effectiveness with an implant-supported mandibular overdenture with bar/clip attachment constructed either from acrylic polymethyl methacrylate (PMMA) resin or flexible thermoplastic material.

Therefore, this trial aimed to compare and evaluate the effects of different complete denture base materials constructed from thermoplastic versus traditional acrylic on the masticatory efficacy of an implant-supported mandibular overdenture with a bar/clip attachment.

## 2. Materials and Methods

Twenty-four completely edentulous patients were selected from the removable prosthodontics clinics at the Faculty of Dental Medicine. The inclusion criteria of this study was a medically fit male patient aged 50–70 years. The patient selection was determined through oral and dental examination, which revealed a Class I arch relationship with adequate interarch space, which was chosen after making a diagnostic cast analysis procedure for each patient. The selected patients were free from any oral hard or soft tissue abnormality that could interfere with the treatment excluding all patients who did not satisfy these normal criteria. Furthermore, written consent detailing all surgical and prosthetic steps of the treatment's advantages and disadvantages was obtained from each patient [18].

In addition, cone beam computerized tomography (CBCT) was performed, guided with a radiographic stent for each selected patient to obtain precise bone height and width measurements at the implant site and accurately determine the size and type of the required implants.

According to the type of complete denture base material's construction, the 24 patients who were selected to participate in this trial were grouped into two groups. Group I (*n* = 12) were the patients with a heat polymerized acrylic resin complete mandibular overdenture heat cured group (HCG), and Group II (*n* = 12) were the patients with a thermoplastic (polyamide) complete mandibular overdenture thermoplastic group (TPG) and both groups were retained by a bar/clip attachment system.

**Figure 1:**
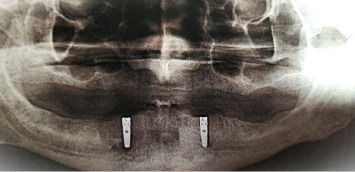
X-ray showing two implants inserted within the interforaminal region of the patient's mandible.

**Figure 2:**
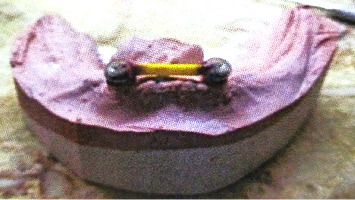
A custom-made bar and clip on the cast to construct a metal cobalt–chromium bar and clip.

**Figure 3:**
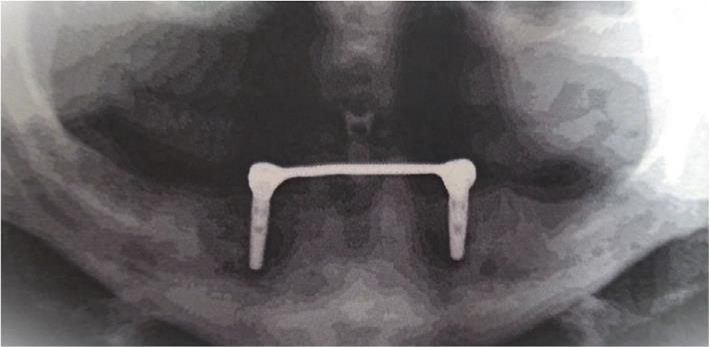
X-ray showing a customized cobalt–chromium metal bar and clip installed for the implants.

All selected and included patients were planned for surgical treatment and submitted to the two-stage surgical protocol of implant placement procedures. Guided by a surgical guide stent, two implants (Dentist, 10 × 3.7 mm) were placed within the inter-oraminal region of the patient's mandible (Figure 1). A routine protocol for postoperative care and maintenance has been established for patients. 3 months after the first phase of surgery. The second phase was completed to expose the implant fixture. A cobalt-chromium metal bar and clip customized for the implants were installed and verified with a panoramic X-ray (Figures 2 and 3). The metal cobalt–chromium custom-made bar and clip were selected to accommodate the available space, cheaper and commonly used.

Regarding the denture construction, the selected patients were delivered a conventional upper complete denture constructed from heat-cured acrylic resin (Acron, GC), while the implanted mandibular arch received a complete overdenture constructed from heat-cured acrylic resin for Group I and a complete thermoplastic denture (Vertex Thermosens, Vertex dental bvj) constructed for Group II. Metal clips were fastened to the fitting surface of the mandibular dentures in both groups before packing the acrylic resin for Group I and before the injection of thermoplastic material for Group II. The finished mandibular implant-supported overdentures were inserted and accurately fitted in the patient's mouth.

In terms of hardness level, the test of masticatory efficiency was performed by examining three different food categories such as apples, bananas, and carrots [19, 20]. Then, each participant installed their mandibular overdentures while sitting upright after being given assurances to help them rest and not be disturbed before eating the test food. The test foods were cut into equal parts measured (1 × 1 cm), and the following measures were recorded using a stopwatch:

• The number of chewing cycle strokes up to the first sallow.
• The number of chewing cycle strokes until the mouth is free of food.
• The number of swallows until the mouth is free of food.
• Time (in seconds) until the mouth is free of food.

After 6-month intervals, clinical evaluations were conducted immediately after dentures were placed. The data were collected from the test groups, and a statistical evaluation was conducted using the SPSS software program (IBM Company). The average, standard deviation, lowest, and highest values were calculated for each group under consideration followed by a one-way analysis of variance (ANOVA) test to ascertain whether any significant differences in the averages of the different groups analyzed. Additionally, the Tukey test was used to evaluate from each other whether the averages differed significantly at the specific probability level *p* < 0.05.

### 2.1. Regulatory Approvals

This clinical trial was ethically approved by Admission Law 903/2935, under the policies and regulations of the Ethical Committee of the Faculty of Dentistry of Al-Azhar University.

## 3. Results

The statistically analyzed data of the number of chewing cycle strokes, swallow, and time in seconds for Group I and Group II were collected and expressed using the mean ± SD value. The single *t*-test was used to compare the tested groups and the threshold of the significant grade fixed at *p* ≤ 0.05.

### 3.1. Number of Chewing Strokes Test up to the First Swallow

**Figure 4:**
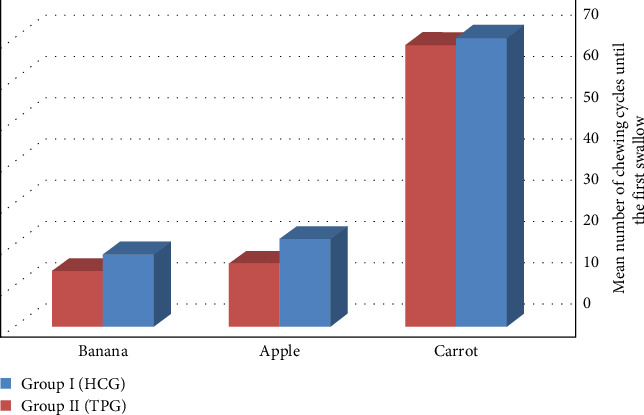
The mean values of the number of chewing cycles until the first swallow between the test groups.

Data from Table 1 showed that Group I recorded a higher number of chewing strokes up to the first swallow of carrot and banana than Group II, with (mean ± SD) values of (70.5 ± 3.5 and 17.5 ± 2.75) for Group I and (68.25 ± 3.5 and 13.5 ± 2.5) for Group II with nonsignificant *p*=0.130 and *p*=0.001, respectively. Controversially, it has been discovered that Group I reported higher numbers of chewing strokes up to the first sallow of apple with a (mean ± SD) value of (21.25 ± 6.75) than Group II which recorded a (mean ± SD) value of (15.25 ± 7.6) with a significant *p*=0.053 (Figure 4).

### 3.2. Number of Chewing Strokes Test until the Mouth Is Free of Food

**Figure 5:**
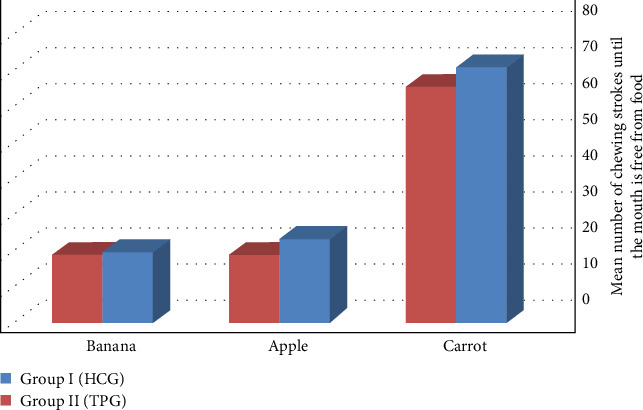
Number of chewing strokes until the mouth is free from food.

Data from Table 2 showed that Group I recorded a higher number of chewing strokes until the mouth was free from apple and banana food than Group II, with (mean ± SD) values of (23.25 ± 6.25 and 19.5 ± 3.5) for Group I and (18.75 ± 6.8 and 18.75 ± 3.9) for Group II with a nonsignificance *p*=0.106 and *p*=0.625, respectively. Controversially, it has been discovered that Group I showed a higher number of chewing strokes before the mouth was free of carrot with (mean ± SD) values of (70.75 ± 5.25) than Group II, which recorded strokes (mean ± SD) value of (65.25 ± 5.3) with a significant *p*=0.018 (Figure 5).

### 3.3. Test of the Number of Swallows until the Mouth Is Free of Food

**Figure 6:**
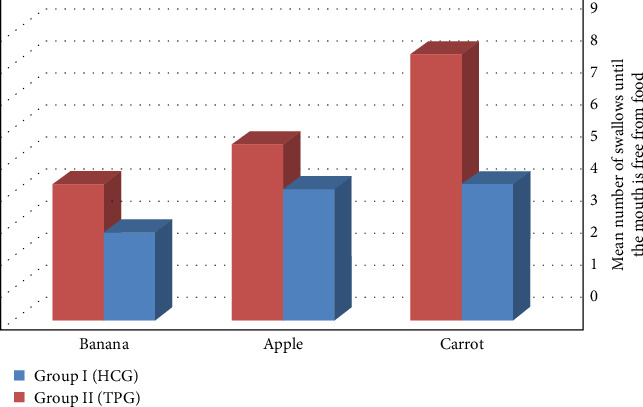
Number of swallows until the mouth is free from food.

Table 3 data showed that Group II recorded higher numbers of swallows before the mouth was free from carrot, apple, and banana food than Group I, with a (mean ± SD) value of (8.3 ± 0.365, 5.5 ± 0.134 and 4.25 ± 0.234) for Group I and (4.25 ± 0.375, 4.1 ± 0.315 and 2.75 ± 0.375) for Group II with a significant *p* ≤ 0.001, *p* ≤ 0.001, and *p* ≤ 0.001, respectively (Figure 6).

### 3.4. Test of Time before the Mouth Is Free of Food in Seconds

**Figure 7:**
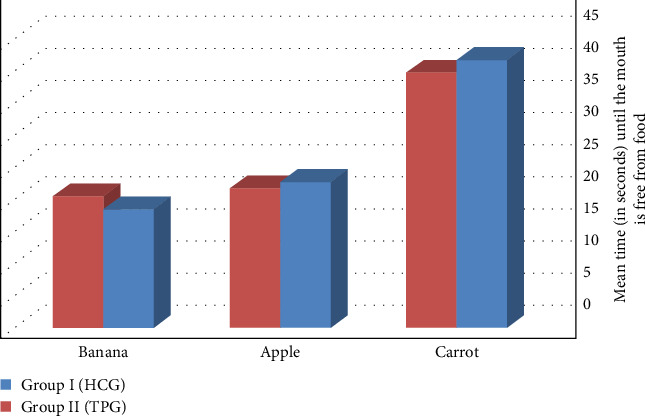
Time (in seconds) until the mouth is free from food.

Data from Table 4 showed that Group I recorded a longer time (in seconds) before the mouth was free from carrot and apple food than Group II, with (mean ± SD) values of (41.75 ± 15.75 and 22.75 ± 4.25) for Group I and (39.75 ± 14.20 and 21.75 ± 3.52) for Group II with nonsignificant *p*=0.7470 and *p*=0.537 respectively. Controversially, it was found that Group II recorded a higher time (in seconds) before the mouth was free of bananas food with (mean ± SD) value of (20.5 ± 2.3) than Group I, which recorded strokes with (mean ± SD) value of (18.5 ± 2.1) with a significant *p*=0.037 (Figure 7).

## 4. Discussion

The results of the current trial disagreed with the null hypothesis which stated there was no variance in the masticatory efficiency of patients treated with implant-supported mandibular overdentures with bar attachment made with acrylic resin or flexible thermoplastic material. By evaluating the masticatory efficiency, the data reported in the study considered and supported the comparison between the thermoplastic overdentures and traditional acrylic overdentures, each on bar attachment systems for implant-retained mandibular overdentures. So, this clinical trial was conducted to compare the masticatory efficiency of implant-supported thermoplastic versus traditional acrylic mandibular overdentures with a custom-made metal bar and clip attachment.

In agreement with Varshney and Bhatia, the two-stage surgical protocol followed in this study started by reflecting the flap, and then, the preparation of implant sites was guided through the surgical stent to avoid angulations or alignment problems that could compromise the force transmission of the final construction [21].

According to Cune et al., the implant system used in this study was selected for its convenience for a submerged two-stage surgical protocol. Brilliant main stability and adequate load dispersion are achieved via the threads of the screws, which increase the implant surface area exposed to bone [22]. Following Gotfredsen and Holm, custom-made bar attachment systems were used, which are fixed in place with fixation screws through the abutments [23].

In agreement with Daher, the conventional and thermoplastic lower dentures were converted into implant-retained overdentures by adjusting their fitting surface to accommodate the attachment system using a method of creating vents within the acrylic base of the dentures with a round stone at low speed to accommodate the metal clips were fitted and attach the denture to the extended bar attachment through clips [24].

In concurrence with Goiato et al.. and Hossain et al., the test foods that were used in this study are four distinct kinds of foods that vary in toughness and consistency (banana, apple, and carrot) which represent soft, crushable, fibrous, and hard types of food while carrot represents a hard, apple as moderate, and banana as a soft food. These foods were used because a clear idea about the effect of the food kind on the corresponding muscular activities during the function can be given [19, 20]. In general, the results of the current clinical trial demonstrated a satisfactory masticatory function in both groups. This is due to excellent retention and stability during the masticatory process from the lower overdenture, as reported by the patients who participated in the study.

A comparison of the masticatory efficiency examined by the four parameters revealed that Group II gives a better result than Group I, with differences between the two groups not being deemed statistically significant for all test food kinds. Except with carrots, the difference is statistically significant for two parameters, which are the number of chewing strokes before the mouth is free of food (*p*=0.018) and the number of swallows before the mouth is free of food (*p* ≤ 0.001). In addition, with apple, the significance is related to two parameters, which are the number of chewing cycles before the first swallow (*p*=0.053) and the number of swallows before the mouth is free (*p* ≤ 0.001). Finally, with bananas, the significance is related to two parameters, which are the number of swallows before the mouth is free of food (*p* ≤ 0.001), as well as the time in seconds before the mouth is free of food (*p*=0.037).

These results are in consequence of those of Bakke et al. and Schimmel et al., who examined patient satisfaction and mastication performance with implant-supported mandibular overdentures. Their research revealed that the mastication time was reduced by using a long bar overdenture. They concluded that the prosthesis appeared to be as successful as the long-bar overdenture because the overdenture significantly decreased the vertical magnitude of the masticatory strokes during all meals, except for the carrot. Furthermore, these results suggest that patients can change their masticatory motions to account for the variations between the two prostheses [25, 26].

For the evaluation of chewing performance, the more effectively food is chewed before swallowing, the more particles are cleaned, and the more effectively food is masticated [19]. Patients found the flexible dentures far more comfortable to wear and fulfilling than the traditional dentures. Our results supported Dhiman RK's research, which attributed its results to the fundamental nature of the denture base materials [27]. Goiato MC et al. came to the same conclusions in their study [28].

In the study of Hazari et al., through masticatory efficiency and performance with complete dentures by recording the total number of chewing cycles and time required to swallow a standardized food item completely, they were reported that a statistically significant number of patients were more satisfied with flexible dentures than with the conventional acrylic dentures because the flexible dentures can be performed better during mastication. These results coincide with the results from this study [29]. Different factors can affect the force of a masticatory bite such as the body mass index, aging, sex, and the type of overdenture material [30, 31].

The clinical limitations of the study from the differences in the quality and quantity of the patient's saliva and the method used to measure the masticatory force. Further research will be recommended to investigate the limits indicated in the study.

## 5. Conclusion

The patients with the thermoplastic overdenture base material supported with bar/clip attachment were more masticatory efficient than the patients with the acrylic resin overdenture base material. The study recommends using overdentures with the base made with the thermoplastic type due to their superior general satisfaction and comfort with swallowing and chewing properties.

## Figures and Tables

**Table 1:** The mean values and standard deviation of the number of chewing cycles until the first swallow between test groups.

Food type	Number of chewing strokes up to the first swallows		*t* student	*p*-Value
	Group I (HCG) (*n* = 12)	Group II (TPG) (*n* = 12)		
Carrot	70.5 ± 3.5	68.25 ± 3.5	1.575	0.130
Apple	21.25 ± 6.75	15.25 ± 7.6	2.045	0.053*⁣*^*∗*^
Banana	17.5 ± 2.75	13.5 ± 2.5	3.728*⁣*^*∗*^	0.001

*Note:* Data were expressed by using mean ± SD.

*t*: Student *t*-test.

*p*: *p*-value for comparing the two studied groups.

*⁣* ^*∗*^Statistically significant at *p* ≤ 0.05.

**Table 2:** The mean values and standard deviations of the number of chewing strokes until the mouth is free of food.

Food type	Number of chewing strokes till the mouth is free of food		*t* student	*p*-Value
	Group I (HCG) (*n* = 12)	Group II (TPG) (*n* = 12)		
Carrot	70.75 ± 5.25	65.25 ± 5.3	2.554*⁣*^*∗*^	0.018*⁣*^*∗*^
Apple	23.25 ± 6.25	18.75 ± 6.8	1.688	0.106
Banana	19.5 ± 3.5	18.75 ± 3.9	0.496	0.625

*Note:* Data were expressed by using mean ± SD.

*t*: Student *t*-test.

*p: p*-value for comparing the two studied groups.

*⁣* ^*∗*^Statistically significant at *p* ≤ 0.5

**Table 3:** The mean values and standard deviations of the number of swallows until the mouth is free of food.

Food type	Number of swallows until the mouth is free of food		*t* student	*p*-Value
	Group I (HCG) (*n* = 12)	Group II (TPG) (*n* = 12)		
Carrot	4.25 ± 0.375	8.3 ± 0.365	26.810*⁣*^*∗*^	<0.001*⁣*^*∗*^
Apple	4.1 ± 0.315	5.5 ± 0.134	14.167*⁣*^*∗*^	<0.001*⁣*^*∗*^
Banana	2.75 ± 0.375	4.25 ± 0.234	11.755*⁣*^*∗*^	<0.001*⁣*^*∗*^

*Note:* Data were expressed by using mean ± SD.

*t*: Student *t*-test.

*p: p*-value for comparing the two studied groups.

*⁣* ^*∗*^Statistically significant at *p* ≤ 0.5.

**Table 4:** The mean values and standard deviations of time (in seconds) until the mouth is free of food.

Food type	Time (in seconds) until the mouth will be free of food		*t* student	*p*-Value
	Group I (HCG) (*n* = 12)	Group II (TPG) (*n* = 12)		
Carrot	41.75 ± 15.75	39.75 ± 14.20	0.327	0.7470
Apple	22.75 ± 4.25	21.75 ± 3.52	0.628	0.537
Banana	18.5 ± 2.1	20.5 ± 2.3	2.225*⁣*^*∗*^	0.037*⁣*^*∗*^

*Note:* Data were expressed by using mean ± SD.

*t*: Student *t*-test.

*p: p*-value for comparing the two studied groups.

*⁣* ^*∗*^Statistically significant at *p* ≤ 0.5.

## Data Availability

The authors confirm that the data supporting the findings of this study are available from the first author upon request.
